# Selective dissolution of halide perovskites as a step towards recycling solar cells

**DOI:** 10.1038/ncomms11735

**Published:** 2016-05-23

**Authors:** Byeong Jo Kim, Dong Hoe Kim, Seung Lee Kwon, So Yeon Park, Zhen Li, Kai Zhu, Hyun Suk Jung

**Affiliations:** 1 School of Advanced Materials Science and Engineering, Sungkyunkwan University, Suwon 16419, Korea; 2 Chemistry and Nanoscience Center, National Renewable Energy Laboratory, Golden, Colorado 80401, USA

## Abstract

Most research on perovskite solar cells has focused on improving power-conversion efficiency and stability. However, if one could refurbish perovskite solar cells, their stability might not be a critical issue. From the perspective of cost effectiveness, if failed, perovskite solar cells could be collected and recycled; reuse of their gold electrodes and transparent conducting glasses could reduce the price per watt of perovskite photovoltaic modules. Herein, we present a simple and effective method for removing the perovskite layer and reusing the mesoporous TiO_2_-coated transparent conducting glass substrate via selective dissolution. We find that the perovskite layer can be easily decomposed in polar aprotic solvents because of the reaction between polar aprotic solvents and Pb^2+^ cations. After 10 cycles of recycling, a mesoporous TiO_2_-coated transparent conducting glass substrate-based perovskite solar cell still shows a constant power-conversion efficiency, thereby demonstrating the possibility of recycling perovskite solar cells.

## Abstract (web summary)

As perovskite-based solar cells are reaching high efficiency, issues of stability and end-of-life become predominant. Here, Kim *et al*. selectively dissolve the perovskite layer and reuse the electron transport layer to fabricate devices with efficiency and stability comparable to those of the original solar cells.

## Graphical abstract

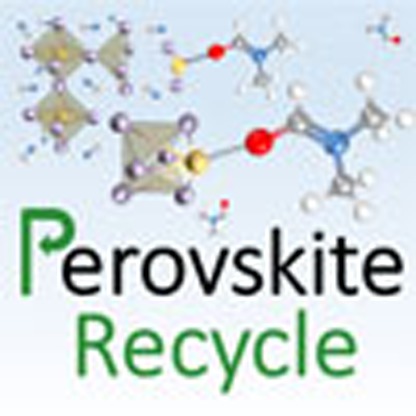

The advent of perovskite solar cells (PSCs) has led to a change in the paradigm of emerging photovoltaic (PV) technology owing to the significant increase in their power-conversion efficiency within the past 3 years^1^,^2^,^3^. Thus far, the best reported efficiency, achieved by Seok and colleagues, is 22.1% (ref. 4). This efficiency is comparable to that of other commercialized solar cells, such as multicrystalline Si (c-Si, 21.3%), cadmium telluride (CdTe, 22.1%) and copper indium gallium selenide (CIGS, 22.3%) solar cells^4^. However, to facilitate commercialization of these emerging PVs, various issues must be addressed, such as long-term stability, the need for Pb-free perovskite light-absorbing materials, the economic feasibility of the manufacturing process and an appropriate ecofriendly disposal process for Pb-contained waste. The price of a Si solar module ranges from $0.6 to $1.0 per Wp (ref. 5), which is significantly higher than the target price of PSCs (lower than $0.2 per Wp, see ref. 6). The target price for PV modules required to achieve grid parity, as determined by the United States Department of Energy, is $0.5 per Wp; therefore, the target figure ($0.2 per Wp) constitutes a paradigm-changing price^7^.

The competitive price of PSCs results from the low material cost and non-vacuum fabrication process of perovskite light absorbers. However, the relatively high material costs for electron- and hole-transport materials, metallic electrode materials (including gold or silver) and transparent conducting glass (TCG) substrate materials render realization of the target price difficult. For example, at the laboratory scale, the unit price of spiro-MeOTAD (2,2',7,7'-tetrakis[N,N-di(4-methoxyphenyl)amino]-9,9'-spirobifluorene), a widely used hole-transport material, is $200 per g (Lumtec). Gold, as a metallic electrode material, and fluorine-doped tin oxide (FTO) glass cost $40 per g and approximately $10 per m^2^, respectively. As such, these expensive materials account for 98% of the costs associated with small, 1 inch × 1 inch square devices. The price of hole-conducting materials is expected to decrease by virtue of new synthesis technologies and large-scale synthesis methods. However, a significant reduction in the prices of gold and FTO glass seems unlikely; in fact, owing to limited mineral resources, their prices may vary significantly depending on global demand.

The recycling of degraded PV modules has been considered as an alternative approach to making PVs economically viable. In particular, improvement of the long-term stability of PSCs is still needed to compete with other types of solar cells, such as c-Si, CdTe and CIGS solar cells, which are being used in commercial PV modules. However, considering the energy payback time (EPBT), which is the time required for a PV module to produce an amount of energy comparable to that consumed during its production, the EPBT of a perovskite PV module is 0.22 years, which is the shortest nominal EPBT compared with all other competitors such as c-Si and CdTe (ref. 8). Therefore, if it were possible to recycle degraded perovskite PV modules, the issues inhibiting the commercialization of perovskite PV modules would be solved, such as their long-term stability, the production of large amounts of Pb-containing waste, the high unit cost of PSCs and the retrieval and reuse of expensive components such as gold, silver and TCG. A shorter payback period of perovskite-based PV modules compared with that of Si-based PV modules could be realized via this process of recycling degraded PSCs.

Therefore, in this paper, we introduce a recycling or repair methodology for PSCs. In this system, trihalide perovskite materials, such as methyl-ammonium lead iodide (CH_3_NH_3_PbI_3_, abbreviated as MALI) as well as formamidinium lead iodide (HC(NH_2_)_2_PbI_3_, abbreviated as FALI) and methyl-ammonium lead bromide (CH_3_NH_3_PbBr_3_, abbreviated as MALBr) mixed halides (in this study, (FALI)_0.85_(MALBr)_0.15_ was used), are removed through a selective dissolution process. This process is capable of dissolving PSCs such that the Au electrodes and the mp-TiO_2_-coated TCG substrates are separated through the dissolution of the perovskite layers. We find that the recycling of PSCs can be realized based on the removal of trihalide perovskite materials and that a small amount of Pb residue has a negligible effect on the ability to reuse the mp-TiO_2_-coated TCG substrate. The selective dissolution mechanism is attributable to the breaking down of the PbI_6_ octahedral frame in the trihalide perovskite owing to the reaction between the partially positive Pb^2+^ ions and a polar aprotic solvent. When the regenerated electron-transport-layer-coated FTO glass (referred to as the mp-TiO_2_-coated TCG substrate in this paper) is reused, the original power-conversion efficiency (15%) is retained over 10 regeneration cycles. This study reveals the potential impact of easily exchangeable PSCs, similar to electric batteries, on the use of PSCs in various applications (for example, as portable power sources in wearable devices and internet-of-things devices).

## Results

### Recycling procedure

**Figure 1:**
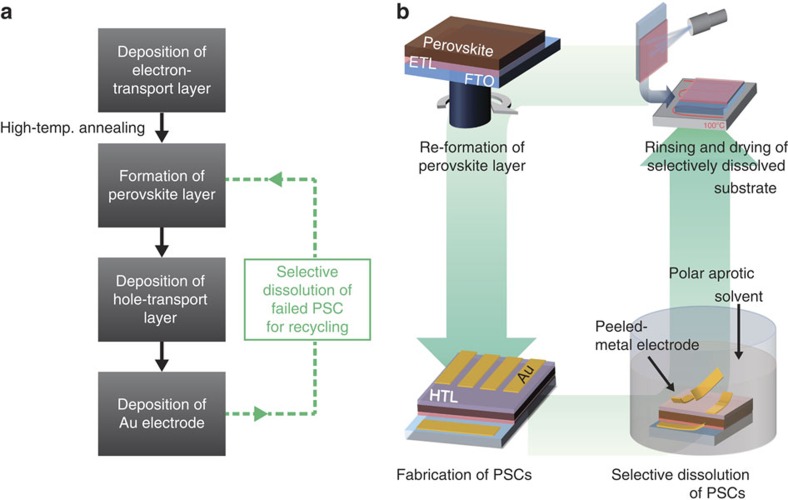
Recycling process for PSCs. (**a**) Flow chart of the fabrication and recycling processes for PSCs. (**b**) Schematic illustration of the detailed process of recycling PSCs via selective dissolution. During soaking in a polar aprotic solvent, the deposited-metal electrode peels away from the device, leaving the clear electron-transport-layer-coated substrate behind, and the hole-transport layer and perovskite layer dissolve.

Figure 1 shows the steps of the recycling process for PSCs. After the deposition and annealing of the electron-transport layer, which is composed (in general) of compact and mp-TiO_2_ layers, the MALI light absorber is coated on the substrate. The hole-transport layer (HTL) and gold electrode are then deposited. During recycling, the MALI perovskite and the HTL dissolve when the fabricated solar cell is immersed in a polar aprotic solvent such as dimethylformamide (DMF), γ-butyrolactone (GBL) or dimethyl sulfoxide (DMSO). The resultant solution contains the recyclable gold electrode and the mp-TiO_2_-coated TCG substrate (Supplementary Fig. 1); the recycling procedure is shown in Supplementary Movie 1. After rinsing and drying of the selectively dissolved mp-TiO_2_-coated TCG substrate, the MALI layer, HTL and gold electrode are re-deposited to yield a recycled PSC.

**Figure 2:**
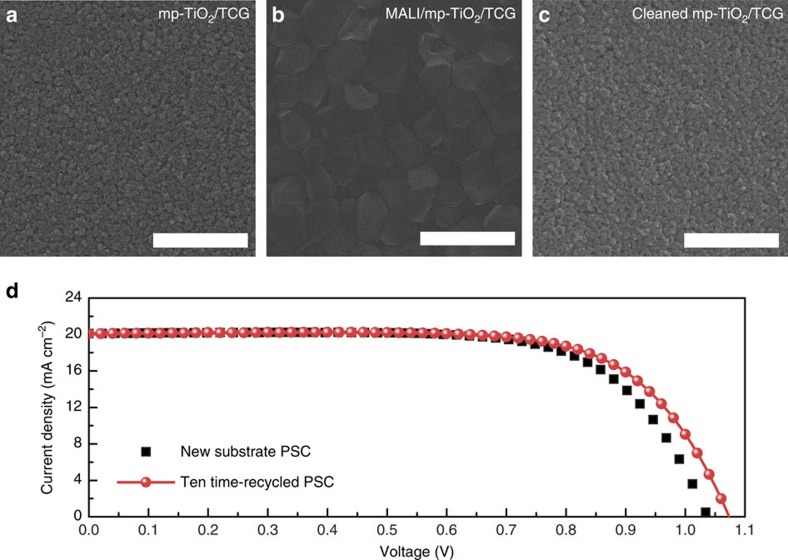
Features of the recycling process performed via selective dissolution. Plane-view FESEM images of (**a**) a new mesoporous TiO_2_ (mp-TiO_2_)/TCG substrate, (**b**) a CH_3_NH_3_PbI_3_ (MALI)-layer-deposited mp-TiO_2_/TCG substrate and (**c**) a cleaned mp-TiO_2_/TCG substrate after the selective dissolution process. For the cleaning of the MALI/mp-TiO_2_/TCG substrate, DMF was used. Scale bars, 1 μm. (**d**) Current density–voltage (*J–V*) curve of a PSC fabricated on a 10-times-recycled mp-TiO_2_/TCG substrate compared with that of a PSC fabricated on a new substrate.

The effect of the polar aprotic solvent on the removal of the MALI perovskite material was determined via field-emission scanning electron microscopy (FESEM). Figure 2a,b show plane-view images of the mp-TiO_2_ layer coated on the TCG substrate and the MALI layer deposited on top of the mp-TiO_2_ layer, respectively. After selective dissolution processing of the MALI-coated substrate using DMF, the MALI layer is easily removed and only the mp-TiO_2_ layer is observed (Fig. 2c). We determined the efficacy of this recycling process by repeatedly recycling (in this work, we performed a total of 10 cycles) the mp-TiO_2_-coated TCG substrate of the PSC. The photocurrent density–voltage (*J–V*) curve of a 10-times-recycled device is similar to that of a PSC based on a new substrate (Fig. 2d and Supplementary Fig. 2). These results indicate that PSCs can be easily recycled.

### Recycling mechanism

**Figure 3:**
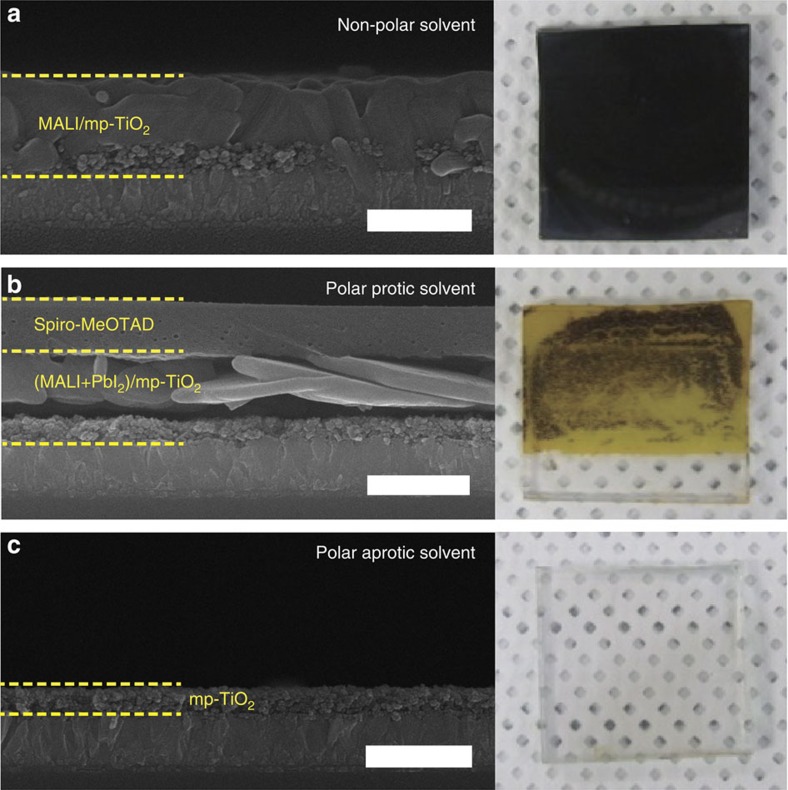
Features of PCSs dissolved in three different types of solvents. Cross-sectional FESEM and photographic images of dissolved PCSs obtained after 30 s of immersion of spiro-MeOTAD/MALI/mp-TiO_2_/TCG in (**a**) non-polar, (**b**) polar protic and (**c**) polar aprotic solvents. Diethyl ether, water and DMF, respectively, were used as the representative solvents. Scale bars, 700 nm.

The selection of an appropriate solvent is essential for an effective recycling process. To investigate the solvent effect, we immersed PSCs in non-polar and protic/aprotic polar solvents. In the case of the non-polar solvents, only the Au/HTL was removed from the Au/HTL/MALI/mp-TiO_2_/TCG substrate, indicating that non-polar solvents selectively dissolve the spiro-MeOTAD layer (Table 1 and Fig. 3). The perovskite layer decomposed in both types of polar solvents, albeit with differing dissolution behaviours. This layer can dissolve in hydrogen-bonded polar protic solvents, such as ethanol or water. However, the dissolution kinetics is quite sluggish compared with the kinetics governing dissolution in a polar aprotic solvent. When the PSCs were immersed in the polar protic solvents, the perovskite layers became yellow in colour and, after more than 24 h, eventually became transparent (Supplementary Fig. 3). This behaviour is attributable to the constituent organic cations (such as the methylamine group) of the perovskite light absorber, which easily dissolve in hydrogen-bonded solvents (for example, H_2_O, ethanol); the low solubility of metal halides (for example, PbI_2_ and PbBr_2_) in polar protic solvents is indicative of the relatively slow S_N_2 reaction compared with that in a polar aprotic solvent^9^,^10^. A polar aprotic solvent can dissolve the perovskite layer completely within a fairly short time after immersion. We tested the dissolution of PSCs in various other solvents, as shown in Supplementary Fig. 4. The results of these attempts were consistent with the results described above.

**Figure 4:**
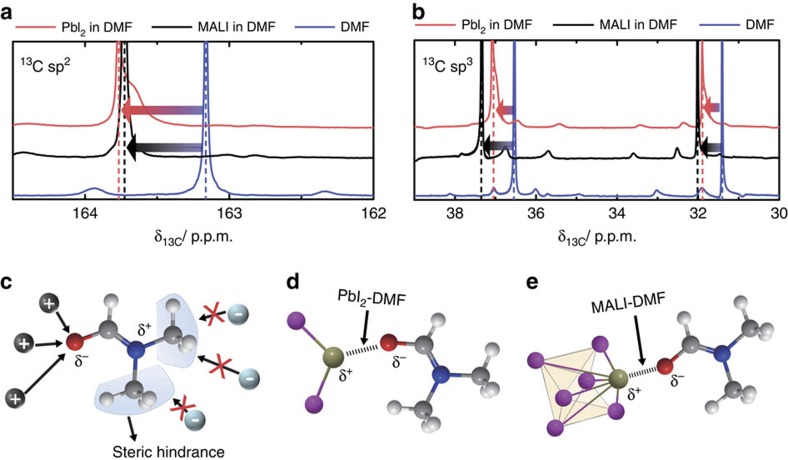
Dissolution mechanism of MALI in a polar aprotic solvent. (**a**) ^13^C sp^2^ and (**b**) ^13^C sp^3^ chemical shifts of PbI_2_–DMF and MALI-DMF away from the corresponding signals for a pure DMF solution. (**c**) Scheme of the resonance structure of DMF, with a partially positive and negative polarity. Chemical bonding structures of the (**d**) PbI_2_ and (**e**) PbI_6_ frames of MALI with the partially negative oxygen of DMF. (Red spheres: oxygen, blue spheres: nitrogen, grey spheres: carbon, white spheres: hydrogen, yellow spheres: lead, violet spheres: iodine.)

To determine whether the bonding between the metal halide and the aprotic solvent plays an important role in the recycling process, ^13^C nuclear magnetic resonance (NMR) measurements were performed at 500 MHz using DMF, 1 M PbI_2_ in DMF and 1 M CH_3_NH_3_PbI_3_ in DMF (the full range of the 500 MHz NMR results is shown in Supplementary Fig. 5a). As Fig. 4a,b show, the main NMR peaks of the PbI_2_–DMF solution (red lines) were shifted downwards compared with those of the DMF solution (blue lines). These results are consistent with the formation of metal halide–DMF bonds; PbI_2_ bonds with the DMF molecule, resulting in the formation of a PbI_2_–DMF structure via the Pb–O bond^9^,^10^,^11^. Because of the resonance structure of DMF (Supplementary Fig. 6), the partially negative oxygen atom in DMF can bond with partially positive atoms, such as Pb^2+^ cations (Fig. 4c). Therefore, aprotic solvents (such as DMF) have the ability to form PbI_2_–DMF compounds, as shown in Fig. 4d. The chemical shifts of the ^13^C sp^2^ and ^13^C sp^3^ signals corresponding to the CH_3_NH_3_PbI_3_–DMF solution (see Fig. 4a,b (black lines)) are similar to those for the PbI_2_–DMF solution. These ^13^C-NMR results indicate that the Pb^2+^ cations in the CH_3_NH_3_PbI_3_ perovskite layer readily bond with the partially negative oxygen atoms in aprotic solvents (Fig. 4e). This bonding enables dissociation of the PbI_6_ octahedral frame that forms the skeleton of the organic–inorganic perovskite material, thereby resulting in high solubility of the perovskite material in polar aprotic solvents.

As shown in Supplementary Movie 1, the used gold electrode can be easily collected during the recycling process. Supplementary Table 1 shows that the collected gold electrodes contained 0.41% lead impurities. The refining of metal collected from electronic and solution wastes is one of the most universal processes for the recycling of metal in industry^12^. Refined gold originating from industrial disposal represents 4% of the entire gold market in the industrial field^13^. Thus, the gold gathered during the recycling process can be easily refined because of its low impurity concentration. Another important problem to be addressed is the removal of lead pollutants from the residual polar aprotic solvent after the recycling process because of the attendant economic and environmental issues. Several methods of removing lead from lead-containing solution wastes have been investigated, such as electrodialysis^14^, solvent extraction^15^, ion exchange^16^ and biosorption^17^. In particular, hydroxyapatite (Ca_10_(PO_4_)_6_(OH)_2_, also called HAP), a biocompatible material present in human bone and teeth, shows a remarkable efficiency for the absorption of Pb^2+^ ions through ion exchange between Ca^2+^ and Pb^2+^ (refs 18, 19). We also demonstrated a lead-removal treatment for residual-lead-containing solution waste via a two-step process combining solvent extraction and ion exchange (Supplementary Fig. 7). In the first step, the lead concentration in a waste solution of 0.05 M MALI in DMF, which was initially 2,017,791.3 p.p.b. (equivalent to μg kg^−1^), was decreased to 28,507.8 p.p.b. during the solvent extraction process. Then, after an ion exchange process with 50 mg HAP, the concentration of residual lead was decreased to 67.4 p.p.b., corresponding to a 99.99% reduction in lead concentration compared with that of the initial solution. Thus, these results show that the proposed two-step lead-removal process is effective in separating lead compounds from residual-lead-containing solution waste.

### Surface analysis of a dissolved mp-TiO_2_-coated FTO substrate

**Figure 5:**
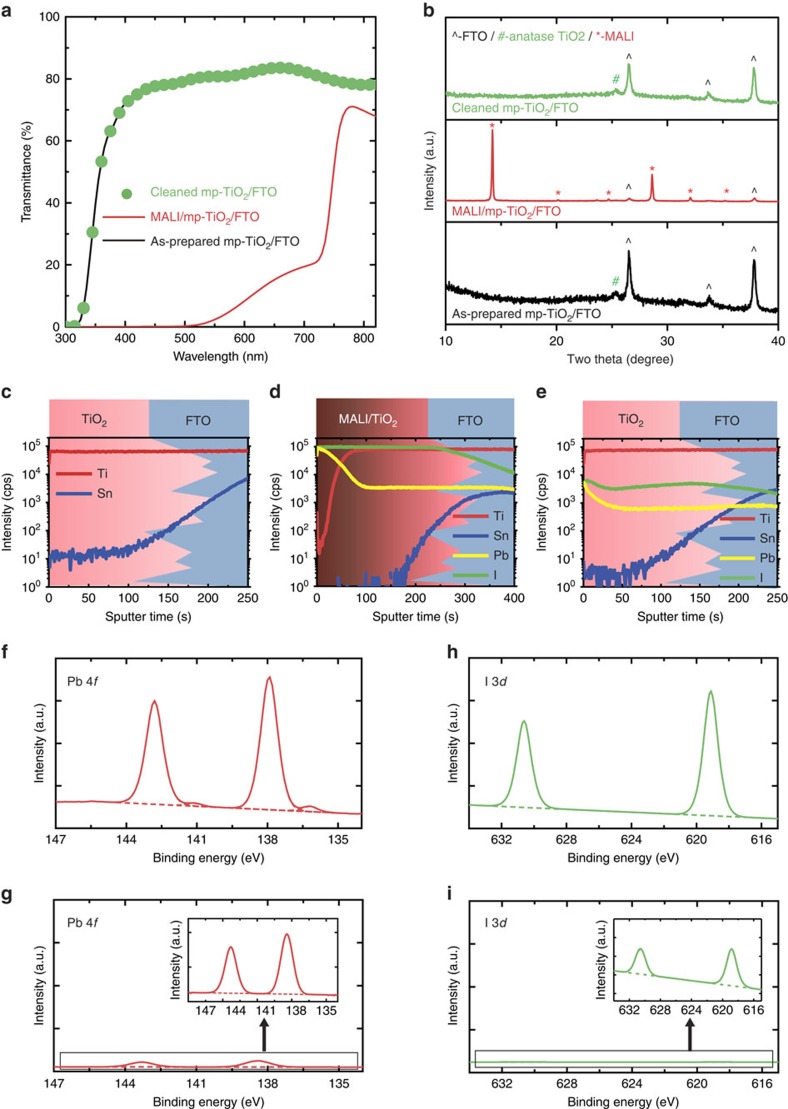
Optical and surface analysis before/after selective dissolution of the substrate. After the recycling process using a polar aprotic solvent, (**a**) the optical transmittance is preserved and (**b**) the X-ray diffraction patterns of the cleaned mp-TiO_2_/FTO substrate exhibit peaks corresponding to anatase TiO_2_ and FTO without MALI or PbI_2_ (^ with black: FTO, # with green: anatase TiO_2_, * with red: MALI). Results of secondary ion mass spectroscopy performed on (**c**) as-prepared mp-TiO_2_/FTO, (**d**) MALI/mp-TiO_2_/FTO and (**e**) cleaned mp-TiO_2_/FTO. The background schematics of the spectra represent the device structure; thus, the initial values of the spectra at 0 s of sputtering time represent each ion concentration at the surface of each substrate. The results for the cleaned mp-TiO_2_/FTO substrate reveal the presence of residual Pb^2+^ and I^-^ ions on the cleaned substrate. X-ray photoemission spectra of (**f**, **g**) Pb 4*f* and (**h**, **i**) I 3*d* on the substrate before and after selective dissolution, respectively. The binding energies of Pb 4*f* and I 3*d* correspond to Pb and I in the perovskite layer and in the form of residual PbI_2_ on the mp-TiO_2_/FTO and cleaned mp-TiO_2_/FTO substrates, respectively.

The optical transmittance spectra of an as-prepared mp-TiO_2_-coated FTO substrate, a MALI/mp-TiO_2_/FTO substrate and a cleaned mp-TiO_2_/FTO substrate are shown in Fig. 5a. The transmittance of the MALI/mp-TiO_2_/FTO substrate is significantly lower than that of the as-prepared mp-TiO_2_/FTO substrate, owing to the large absorption coefficient of the MALI layer. After the perovskite layer is removed, the transmittance is restored to that of the as-prepared substrate, indicating that perovskite-layer removal and the dissolution process do not affect the optical properties of the substrate. Figure 5b shows the X-ray diffraction patterns of the films^20^. The peaks in the pattern of the DMF-cleaned mp-TiO_2_/FTO substrate correspond only to anatase TiO_2_ and FTO, indicating that the perovskite layer was removed by the DMF solvent. The surface state of the cleaned substrate was determined from atomic depth profiles obtained via secondary ion mass spectrometry (SIMS) for each film. As Fig. 5d shows, Pb and I were present in the MALI/mp-TiO_2_/FTO substrate. The X-ray diffraction pattern of the sample (Fig. 5b) indicates that these elements are components of the MALI perovskite material. These were detected even after the perovskite layer was removed, indicating that residual Pb^2+^ and I^−^ions (not detected via X-ray diffraction) were present on the surface of the mp-TiO_2_ layer (Fig. 5e). Supplementary Fig. 8 shows the X-ray photoemission spectra of the films. Consistent with the X-ray diffraction and SIMS data, the presence of Pb and I peaks in the signal from the pre-dissolution sample indicates that a perovskite layer formed on the MALI/mp-TiO_2_/FTO substrate.

The X-ray photoemission spectra of the cleaned mp-TiO_2_/FTO substrate also contain Pb and I peaks. Figure 5f,h and Fig. 5g,i show the X-ray photoemission spectra of the MALI/mp-TiO_2_/FTO substrate and the cleaned mp-TiO_2_/FTO (inset) substrate, respectively. The binding energies of Pb 4*f* (142.8 and 137.9 eV) and I 3*d* (630.6 and 619.1 eV), shown in Fig. 5f,h, respectively, indicate that the Pb and I ions were incorporated into the MALI perovskite structure^21^,^22^. The peaks in Fig. 5g,i corresponding to the binding energies of Pb and I ions remaining on the surface of the cleaned mp-TiO_2_/FTO substrate appear in positions that are typical of PbI_2_ (refs 23, 24).

We also investigated the surface states of cleaned mp-TiO_2_/FTO substrates that had been selectively dissolved with various cleaning times (from 1 to 10 min) and numbers of recycling cycles (from one to five cycles with 30 s of rinsing between each cycle) for complete removal of residual PbI_2_ and confirmation of the accumulation of residual PbI_2_, respectively. Supplementary Fig. 9a,b show that the intensities of the Pb 4*f* peak for cleaned mp-TiO_2_/FTO substrates treated with various cleaning times (from 1 to 10 min) and in multiple cleaning cycles (from one to four cycles) were of similar intensity regardless of the cleaning conditions. Furthermore, the Pb 4*f* peak of the five-times-recycled mp-TiO_2_/FTO also showed a similar intensity to that of the once-recycled substrate (Supplementary Fig. 9c). It appears impossible to remove the residual PbI_2_ completely, but the amount of residual PbI_2_ on the TiO_2_ surface was negligible compared with that in the original MALI layer, and no accumulation of residual PbI_2_ occurred under repeated recycling.

Other groups have investigated the effect of non-stoichiometric MAI/PbI_2_ molar ratios (of PbI_2_ and CH_3_NH_3_I (MAI)) on perovskite crystal growth or crystallinity, leading to improved PV properties; molar ratios ranging from 0.83 to 1.2 were considered in those studies^25^,^26^. In addition, Cao *et al*. have reported that residual PbI_2_, which forms between the perovskite and the TiO_2_, acts as a charge-recombination-hindering layer^27^. No such phenomenon was observed in the present study, in which the performance of the recycled PSCs remained stable, even in the presence of PbI_2_. Therefore, a polar aprotic solvent is capable of dissolving MALI perovskite materials within a rather short amount of time, although the PbI_2_ on the TiO_2_ surface is only partially dissolved. The residual PbI_2_ layer does not affect the PV properties of the regenerated PSC, thus enabling the recycling of these cells.

## Discussion

**Figure 6:**
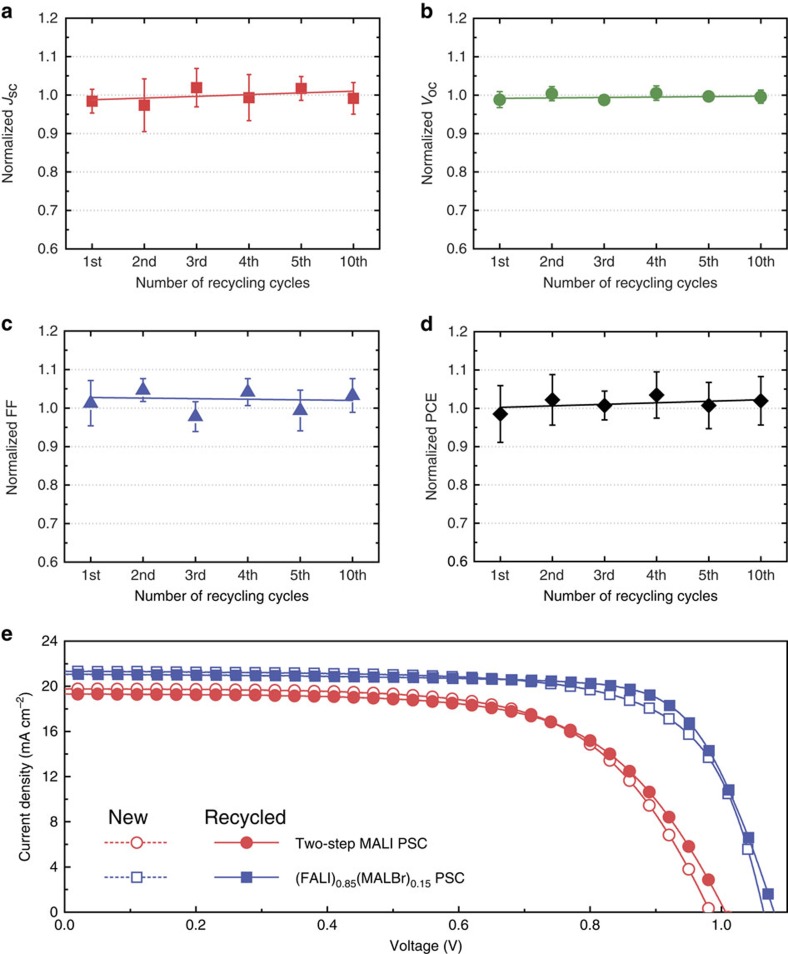
Photovoltaic performance characteristics of recycled PSCs. Normalized trends in the (**a**) short-circuit current density, (**b**) open-circuit voltage, (**c**) fill factor and (**d**) efficiency of MALI-based PSCs fabricated on 1st-, 2nd-, 3rd-, 4th-, 5th- and 10th-time recycled substrates compared with those of the MALI-PSC fabricated on the newly prepared substrate. (**e**) *J–V* curves for two-step processed MALI-based PSCs (red spheres) and (FALI)_0.85_(MALBr)_0.15_-based PSCs (blue squares) on new (dotted lines and empty symbols) and once-recycled (solid lines and filled symbols) mp-TiO_2_/TCG substrates. The error bars in **a**–**d** indicate the s.d.'s calculated from five samples.

The nearly constant PV performance observed during the 10-times-repeated recycling of the mp-TiO_2_/FTO substrate (see Fig. 6) demonstrates the recyclability of PSCs using the proposed method. Furthermore, a recycled MALI-PSC, fabricated using a cleaned mp-TiO_2_/FTO substrate recovered from a degraded MALI-PSC through selective dissolution, exhibited a PV performance comparable to that of the MALI-PSC based on the fresh mp-TiO_2_/FTO substrate (Supplementary Fig. 10). Four individual cells were measured for each type of device to investigate the influence of the recycling process on long-term stability. The recycled MALI-PSC device showed stability comparable to that of the fresh MALI-PSC device (Supplementary Fig. 11 and Supplementary Table 2). To confirm the feasibility of the recycling process for various fabrication processes, such as a two-step process, and for different compositions, such as formamidinium- or bromide-containing perovskite, we fabricated two-step MALI- and (FALI)_0.85_(MALBr)_0.15_-based PSCs using our selective dissolution recycling process. The results presented in Fig. 6e show that the selective dissolution process is also compatible with other perovskite fabrication processes and with PSCs based on different compositions. These results clearly demonstrate that our recycling process does not influence the PV performance and that it is generally applicable to PSCs of any kind prepared with different perovskite deposition processes and compositions. We also found that the recycling process is applicable to planar-structured and flexible PSCs (Supplementary Fig. 12).

These results may change the current paradigm in the field of solar cells and their application. Although the stability of PSCs remains an important issue, the long-term stability of PSCs is not as essential as it is in the case of commercialized solar cells. If PSCs can be easily refurbished, then these cells can be used as batteries that could become available in consumer products. The combination of high efficiency, recyclability and plastic-based flexibility in PSC technology will shift the paradigm of utilization of perovskite-based PV devices.

## Methods

### Materials

All materials, except for 1-butanol (Tokyo Chemical Industry), were purchased from Sigma-Aldrich. All materials were used as received. Methyl-ammonium iodide (CH_3_NH_3_I, MAI) was synthesized using a previously reported method^28^. A solution of 57 wt.% hydriodic acid in water and a solution of 33 wt.% methylamine (CH_3_NH_2_) in absolute ethanol were stirred into 100 ml of ethanol at room temperature. The precipitate, methylamine iodide, was obtained using a rotary evaporator. Formamidinium iodide (HC(NH_2_)_2_I, FAI) was prepared using a previously reported method^29^, in which 25.2 g and 48 ml of formamidine acetate and hydriodic acid, respectively, were mixed into 250 ml of methanol and reacted at room temperature for 2 h. The resulting precipitate was collected using a rotary evaporator. The synthesized MAI and FAI were washed with diethyl ether and then recrystallized from ethanol and dried in a vacuum oven overnight.

### Recycling process

The selective dissolution process for recycling mp-TiO_2_/TCG substrates that have been used in PSCs consisted of several simple steps. In the first step, the trihalide perovskite layer and spiro-MeOTAD were removed by immersing the PSCs in a polar aprotic solvent, such as DMF, GBL or DMSO. The PSCs were then shaken for 30 s in solution. The cleaned mp-TiO_2_/TCG substrates were rinsed with deionized water and then dried on a hot plate. For optimal performance, the cleaned substrates were annealed at 500 °C for 1 h. The gold (Au) material in the PSCs does not react with polar aprotic solvents, and, therefore, the gold that remained in the solutions after cleaning could be collected.

The removal of lead from a polar aprotic solvent used for the recycling process was performed via a two-step process combining solvent extraction with ion exchange. The prepared solution of 0.05 M MALI in DMF was poured into ether, a non-polar solvent. The precipitated lead compound was separated via centrifugation at 8,000 r.p.m. for 10 min. A laboratory-prepared HAP powder (between 10 and 50 mg) was added to the remaining solution to remove residual Pb^2+^ ions via ion exchange (from Ca^2+^ to Pb^2+^). After the addition of the HAP powder, the solution was stirred at 200 r.p.m. for 3 h. After sufficient stirring, the Pb^2+^-ion-adsorbed HAP powder was separated using a centrifuge at 8,000 r.p.m. for 10 min. The solution remaining after the completion of each step was analysed via inductively coupled plasma-mass spectrometry (Perkin-Elmer SCIEX, NexION). All solutions were analysed three times, and the results were averaged.

### Synthesis of hydroxyapatite

HAP with a high surface area was synthesized using a hydrothermal method based on a previous report by Jiang *et al*.^30^. In a typical synthesis procedure, 2 mmol of CaCl_2_˙2H_2_O (Sigma-Aldrich, ⩾99%) and 1 g of poly-L-aspartic acid sodium salt (Sigma-Aldrich, mol. wt. 5,000–15,000) was dissolved in 20 ml of deionized water under 30 min of stirring (solution 1). Then, 1.2 mmol of (NH_4_)_2_HPO_4_ (Sigma-Aldrich, ⩾98%) was dissolved in 15 ml of deionized water under vigorous stirring for 30 min (solution 2). After stirring, solution 2 was poured into solution 1. The mixed solution was sealed in a 50-ml Teflon liner, placed in a stainless steel autoclave and maintained at 200 °C for 12 h. The precipitated HAP was washed with distilled water and ethanol. After washing, the obtained HAP powder was dried in a freeze dryer for 12 h.

### Device fabrication

FTO-coated glass substrates (Pilkington TEC15) were cleaned for 15 min with acetone, ethanol and DI water in an ultrasonic bath. A compact hole-blocking layer of TiO_2_ was spin-coated (at 3,000 r.p.m. for 20 s) on the substrate, using 0.15 M titanium diisopropoxide bis(acetylacetonate) (75 wt.% in isopropanol) in anhydrous 1-butanol. This layer was then baked at 130 °C for 5 min. An ∼200-nm-thick layer of porous mp-TiO_2_ was prepared on top of the TiO_2_ layer using a diluted TiO_2_ paste (EtOH:Dyesol 18NRT TiO_2_ paste in a ratio of 4.5:1) and calcining at 500 °C for 1 h. The resulting mp-TiO_2_/FTO substrate was immersed for 15 min in a 0.04 M TiCl_4_ solution at 70 °C, rinsed with DI water and then annealed at 500 °C for 30 min.

The prepared CH_3_NH_3_PbI_3_ solution (46 wt.% CH_3_NH_3_I and PbI_2_, at an equal molar ratio, in a mixture of GBL and DMSO (7:3 *v*/*v*)) was spin-coated at 1,000 and 5,000 r.p.m. for 10 and 30 s, respectively. During the 5,000-r.p.m. step, 1 ml of a toluene solution was dropped on the spinning CH_3_NH_3_PbI_3_ solution. The spin-coated substrate was then dried at 130 °C for 20 min (ref. 31). Subsequently, 30 μl of a HTL solution (72 mg of spiro-MeOTAD, 28.8 μl of 4-tert-butylpyridine and 17.6 μl of Li-TFSI solution (720 mg of Li-TFSI in acetonitrile) in 1 ml of chlorobenzene) was formed via spin-coating at 4,000 r.p.m. for 30 s. The Au electrodes were deposited on the PSCs via thermal evaporation. The substrate size and the active device area were 2 × 2 and 0.14 cm^2^, respectively.

### Device characterization

The surface morphology and cross-sectional structure were examined via FESEM (JSM-7600F, JEOL). ^13^C-NMR measurements were performed using a 500-MHz NMR spectrometer (Avance-500, Bruker). In addition, the transmittance spectra of the substrate and the perovskite-coated substrate were collected via UV–vis/NIR spectrometry (Cary 5000, Agilent Technologies). The crystalline structure was determined using an X-ray diffractometer (New D8 Advanced, Bruker). Furthermore, compositional depth profiles of the pristine, perovskite-coated and cleaned substrates were obtained via time-of-flight SIMS (ION-TOF). X-ray photoelectron spectroscopy (X-ray photoemission spectra, Sigma Probe, ThermoVG) was used to determine the chemical states of Pb and I on the surfaces of the substrates. The corresponding spectra were acquired using monochromatic A1-Kα radiation (100 W) and were calibrated with respect to the C 1-s level (at 284.5 eV). Moreover, the PV properties were measured using a solar simulator (Newport Oriel Solar 3A Class AAA, 64023A) equipped with a 450-W xenon lamp (Newport 6279NS) and a potentiostat (CHI 600D, CH Instruments). The light intensity was adjusted using a standard Si solar cell (Oriel, VLSI standards; 1 sun is equivalent to 100 mA cm^−2^).

### Data availability

The authors declare that the data supporting the findings of this study are available within the article and its Supplementary Information files.

## Additional information

**How to cite this article:** Kim, B.J. *et al*. Selective dissolution of halide perovskites as a step towards recycling solar cells. *Nat. Commun.* 7:11735 doi: 10.1038/ncomms11735 (2016).

## Supplementary Material

Supplementary Information Supplementary Figures 1-12 and Supplementary Tables 1-2

Supplementary Movie 1 This movie provides a real time play of selective dissolution process for recycling of perovskite solar cells. Also, it shows the possibility of reusing gold electrode after recycling process.

## Figures and Tables

**Table 1:** Various solvents used for the selective dissolution of the perovskite layer and their dipole moments.

**Solvent**	**Chemical formula**	**Dipole moment (D)**
*Non-polar solvents*		
Toluene	C_6_H_5_-CH_3_	0.375
Diethyl ether	CH_3_-CH_2_-O-CH_2_-CH_3_	1.15
Dichoromethane	CH_2_Cl_2_	1.60
Chlorobenzene	C_6_H_5_-Cl	1.69
		
*Polar protic solvents*		
2-Propanol	CH_3_-CH(-OH)-CH_3_	1.58
Ethanol	CH_3_-CH_2_-OH	1.69
Methanol	CH_3_-OH	1.70
Water	H-O-H	1.85
		
*Polar aprotic solvents*		
Acetone	CH_3_-C(=O)-CH_3_	2.88
Dimethylformamide	H-C(=O)N(CH_3_)_2_	3.82
Acetonitrile	CH_3_-C≡N	3.92
Dimethyl sulfoxide	CH_3_-S(=O)-CH_3_	3.96
γ-Butyrolactone	/-C_2_H_4_-O-C_2_H_4_-\=O	4.27

## References

[b1] Kim H.-S. . Lead iodide perovskite sensitized all-solid-state submicron thin film mesoscopic solar cell with efficiency exceeding 9%. Sci. Rep. 2, 591 (2012). 22912919 10.1038/srep00591 PMC3423636

[b2] Burschka J. . Sequential deposition as a route to high-performance perovskite-sensitized solar cells. Nature 499, 316–319 (2013). 23842493 10.1038/nature12340

[b3] Zhou H. . Interface engineering of highly efficient perovskite solar cells. Science 345, 542–546 (2014). 25082698 10.1126/science.1254050

[b4] NREL, Best Research-Cell Efficiencies http://www.nrel.gov/ncpv/images/efficiency_chart.jpg (2015).

[b5] Metz A. . International Technology Roadmap for Photovoltaic (ITRPV.net) Results 2014 http://www.itrpv.net/Reports/Downloads/2015/ (2015).

[b6] Savage L. Perovskite photovoltaics: hitting their stride. Opt. Photon. News 25, 26–33 (2014).

[b7] U.S. Department of Energy. *SunShot Vision Study. Report No. DOE/GO-102012-3037.* http://www.osa-opn.org/opn/media/Images/PDF/2014/1114/26-33_Perovskite-Nov.pdf?ext=.pdf ( Department of Energy (2012).

[b8] Gong J. . Perovskite photovoltaics: life-cycle assessment of energy and environmental impacts. Energy Environ. Sci. 8, 1953–1968 (2015).

[b9] Miller J. . Dipolar aprotic solvents in bimoleculer aromatic nucleophilic substitution reactions. J. Am. Chem. Soc. 83, 117–123 (1961).

[b10] Parker A. J. The effects of solvation on the properties of anions in dipolar aprotic solvents. Q. Rev. Chem. Soc. 16, 163–187 (1962).

[b11] Wakamiya A. . Reproducible fabrication of efficient perovskite-based solar cells: x-ray crystallographic studies on the formation of CH_3_NH_3_PbI_3_ layers. Chem. Lett. 43, 711–713 (2014).

[b12] Cui J. . Metallurgical recovery of metals from electronic waste: a review. J. Hazard. Mater. 158, 228–256 (2008). 18359555 10.1016/j.jhazmat.2008.02.001

[b13] Hagelüken C. . Recycling of gold from electronics: cost-effective use through ‘Design for Recycling. Gold Bull. 43, 209–220 (2010).

[b14] Mohammadi T. . Effect of operating parameters on Pb^2+^ separation from wastewater using electrodialysis. Desalination 167, 379–385 (2004).

[b15] Shah D. B. . Lead removal from foundry waste by solvent extraction. J. Air Waste Manage. Assoc. 45, 150–155 (1995). 10.1080/10473289.1995.10467354 15658154

[b16] Ahmed S. . The removal of cadmium and lead from aqueous solution by ion exchange with Na-Y zeolite. Sep. Purif. Technol. 13, 57–64 (1998).

[b17] Axtell N. R. . Lead and nickel removal using microspora and lemna minor. Bioresour. Technol. 89, 41–48 (2003). 12676499 10.1016/s0960-8524(03)00034-8

[b18] Cui L. . Mechanism of Pb(II) and methylene blue adsorption onto magnetic carbonate hydroxyapatite/graphene oxide. RSC Adv. 5, 9759–9770 (2015).

[b19] Lei Y. . Bioinspired fabrication and lead adsorption property of nano-hydroxyapatite/chitosan porous materials. RSC Adv. 5, 98783–98795 (2015).

[b20] Kutes Y. . Direct observation of ferroelectric domains in solution-processed CH _3_NH_3_PbI_3_ perovskite thin films. J. Phys. Chem. Lett. 5, 3335–3339 (2014). 26278441 10.1021/jz501697b

[b21] Lindblad R. . Electronic structure of TiO_2_/CH_3_NH_3_PbI_3_ perovskite solar cell interfaces. J. Phys. Chem. Lett. 5, 648–653 (2014). 26270831 10.1021/jz402749f

[b22] Conings B. . Perovskite-based hybrid solar cells exceeding 10% efficiency with high reproducibility using a thin film sandwich approach. Adv. Mater. 26, 2041–2046 (2013). 24338932 10.1002/adma.201304803

[b23] Roy A. . Spectroscopic studies on quantum dots of PbI_2_. Spectrochim. Acta A Mol. Biomol. Spectrsc. 48, 1779–1787 (1992).

[b24] Moulder J. F. . Handbook of X-ray Photoelectron Spectroscopy: a Reference Book of Standard Spectra for Identification and Interpretation of XPS Data Physical Electronics Division Perkin-Elmer Corp (1995).

[b25] Roldán-Carmona C. . High efficiency methylammonium lead triiodide perovskite solar cells: the relevance of non-stoichiometric precursors. Energy Environ. Sci. 8, 3550–3556 (2015).

[b26] Yang M. . Square-centimeter solution-processed planar CH_3_NH_3_PbI_3_ perovskite solar cells with efficiency exceeding 15%. Adv. Mater. 27, 6363–6370 (2015). 26414514 10.1002/adma.201502586

[b27] Cao D. H. . Remnant PbI_2_, an unforeseen necessity in high-efficiency hybrid perovskite-based solar cells? APL Mater. 2, 091101–091107 (2014).

[b28] Lee M. M. . Efficient hybrid solar cells based on meso-superstructured organometal halide perovskites. Science 338, 643–647 (2012). 23042296 10.1126/science.1228604

[b29] Wozny S. . Controlled humidity study on the formation of higher efficiency formamidinium lead triiodide-based solar cells. Chem. Mater. 27, 4814–4820 (2015).

[b30] Jiang S.-D. . Fabrication of hydroxyapatite hierarchical hollow microspheres and potential application in water treatment. J. Phys. Chem. C. 116, 4484–4492 (2012).

[b31] Jeon N. J. . Solvent engineering for high-performance inorganic–organic hybrid perovskite solar cells. Nat. Mater. 13, 897–903 (2014). 24997740 10.1038/nmat4014

