# Supplementary material for: Selective dissolution of halide perovskites as a step towards recycling solar cells
Source: Nat Commun. 2016 May 23;7:11735. doi: 10.1038/ncomms11735 (PMC4879253; doi:10.1038/ncomms11735)
Supplement: Supplementary Information — Supplementary Figures 1-12 and Supplementary Tables 1-2 [file ncomms11735-s1.pdf]

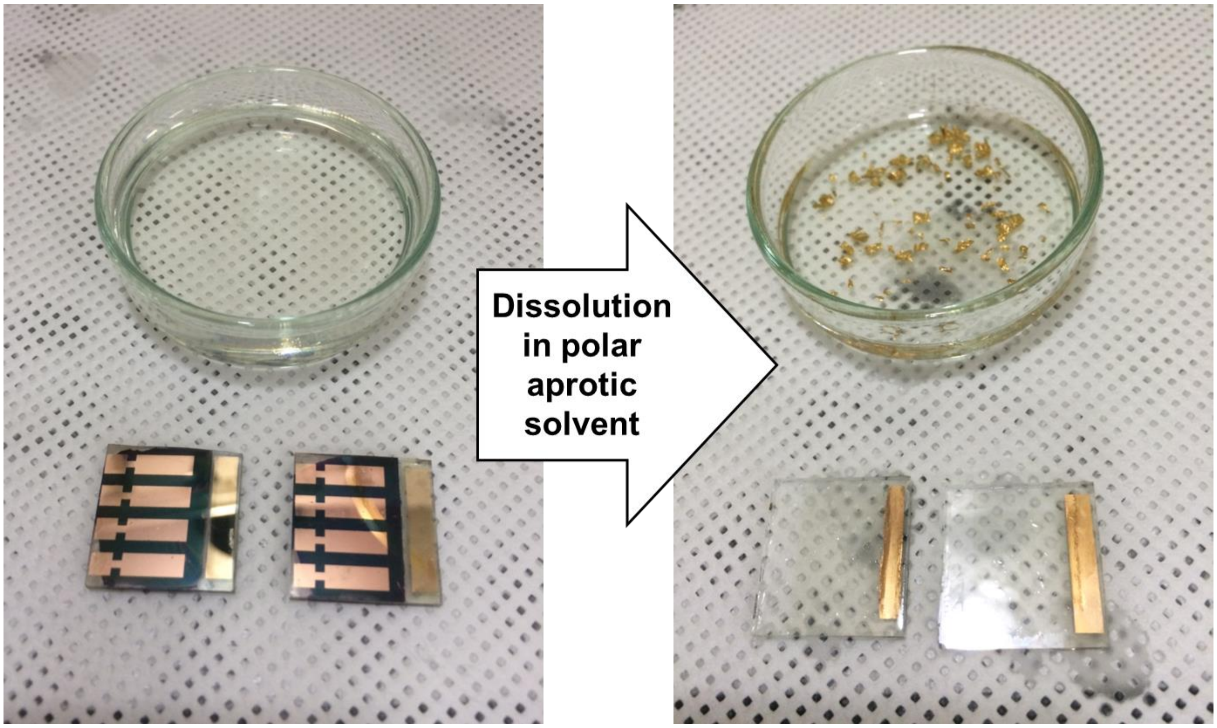

Supplementary Figure 1. Optical images before/after selectively dissolution the perovskite solar cells. The manufactured perovskite layer is easily dissolved in a polar aprotic solvent (dimethylformamide, DMF, was used in this study). The cleaned mp-TiO_2/transparent conducting glass (TCG) substrate and the expensive gold electrode can be collected and recycled.

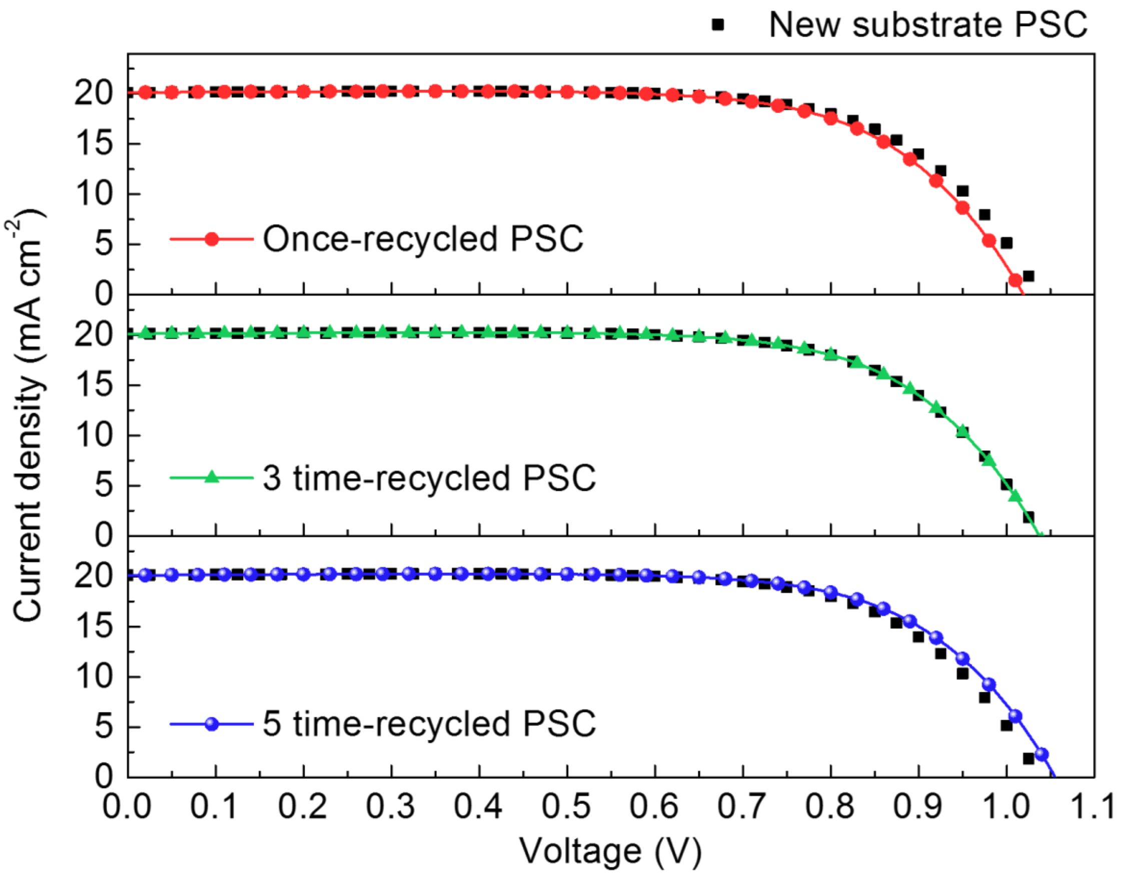

Supplementary Figure 2. Current density-voltage curve of $CH_3NH_3PbI_3$ (MALI)-perovskite solar cells (PSCs) on the recycled substrate. The photovoltaic performance of MALI-PSC on the 1st, 3rd and 5th uses of the recycled mp-TiO_2-coated TCG substrate clearly exhibits that the recycling process does not affect PSC efficiency.

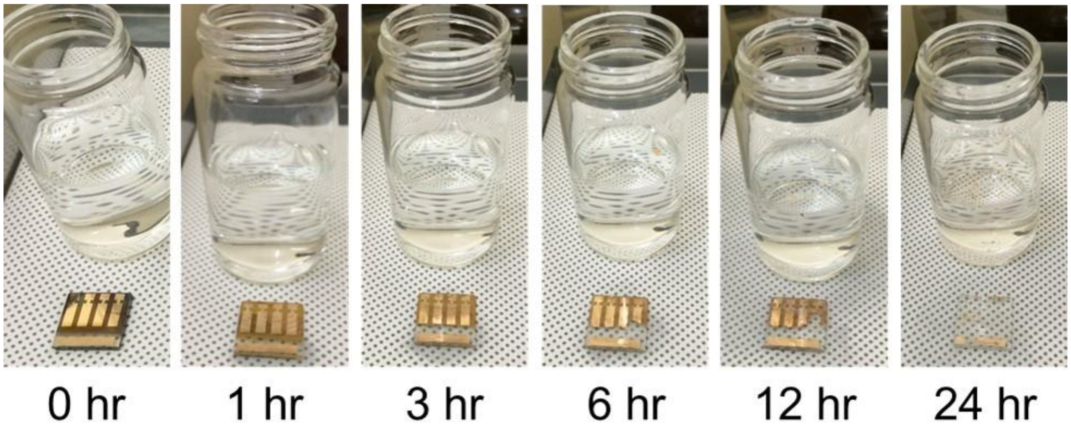

Supplementary Figure 3. Recycle procedure of PSC using DI water. A polar protic solvent is capable of dissolving the organic-inorganic perovskite layer. However, a longer time was required to dissolve the perovskite layer compared to using a polar aprotic solvent. At the early time points, the perovskite layer turned a yellowish color (see Table 1, in the case of the polar protic solvent) and then became transparent.

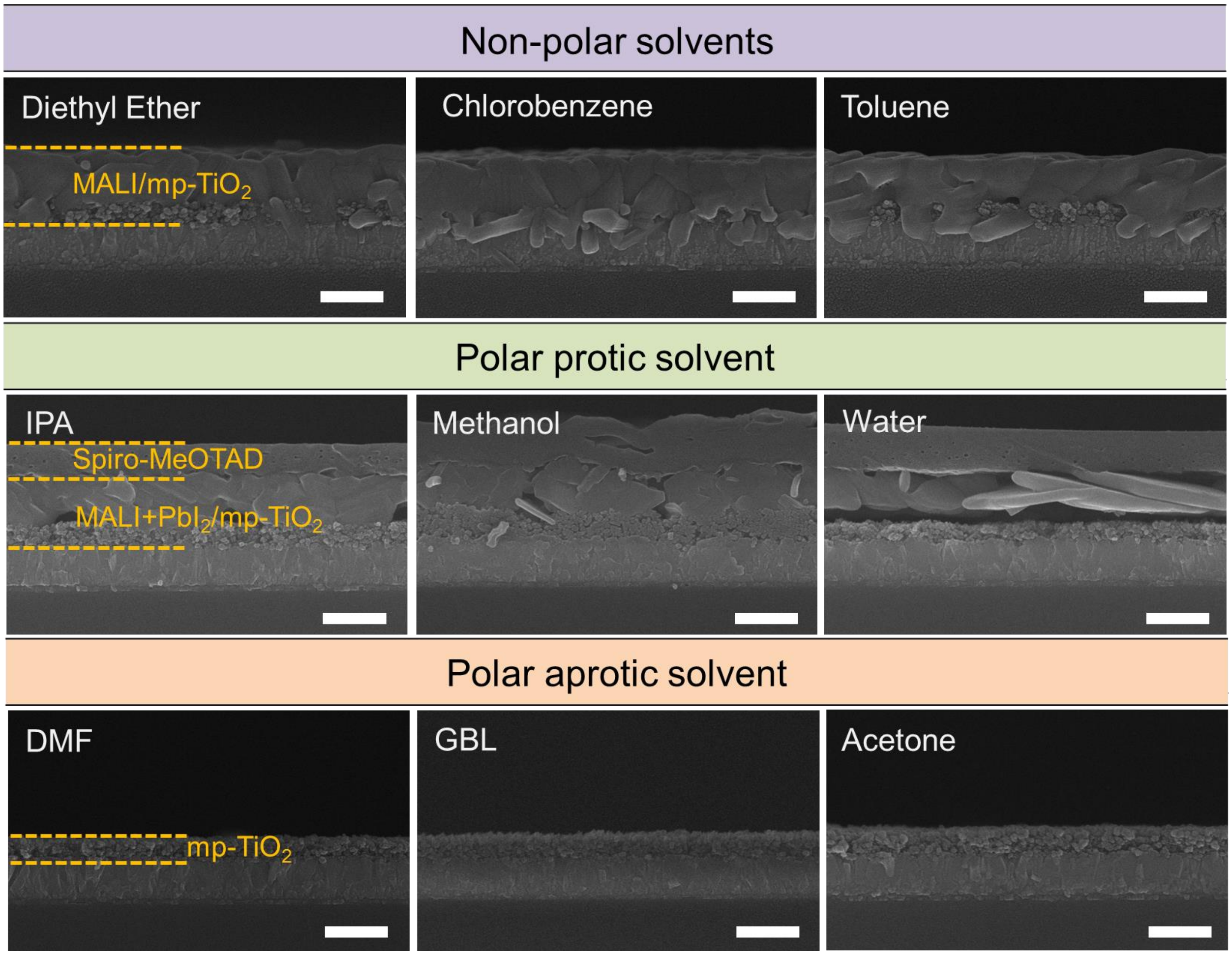

Supplementary Figure 4. Cross-section FESEM images after a 30 sec-immersion of Spiro-MeOTAD/MALI/mp-TiO_2/TCG into the various solvents. Only the Spiro-MeOTAD (2,2',7,7'-Tetrakis[N,N-di(4-methoxyphenyl)amino]-9,9'-spirobifluorene) of the hole-transport layer solution was removed in the various non polar solvents. The MALI layer was partially decomposed in the polar protic solvent; however, the Spiro layer remained on the decomposed MALI layer. In the case of using polar aprotic solvents, the MALI layer and Spiro-MeOTAD were completely dissolved. The scale bar represents 500 nm.

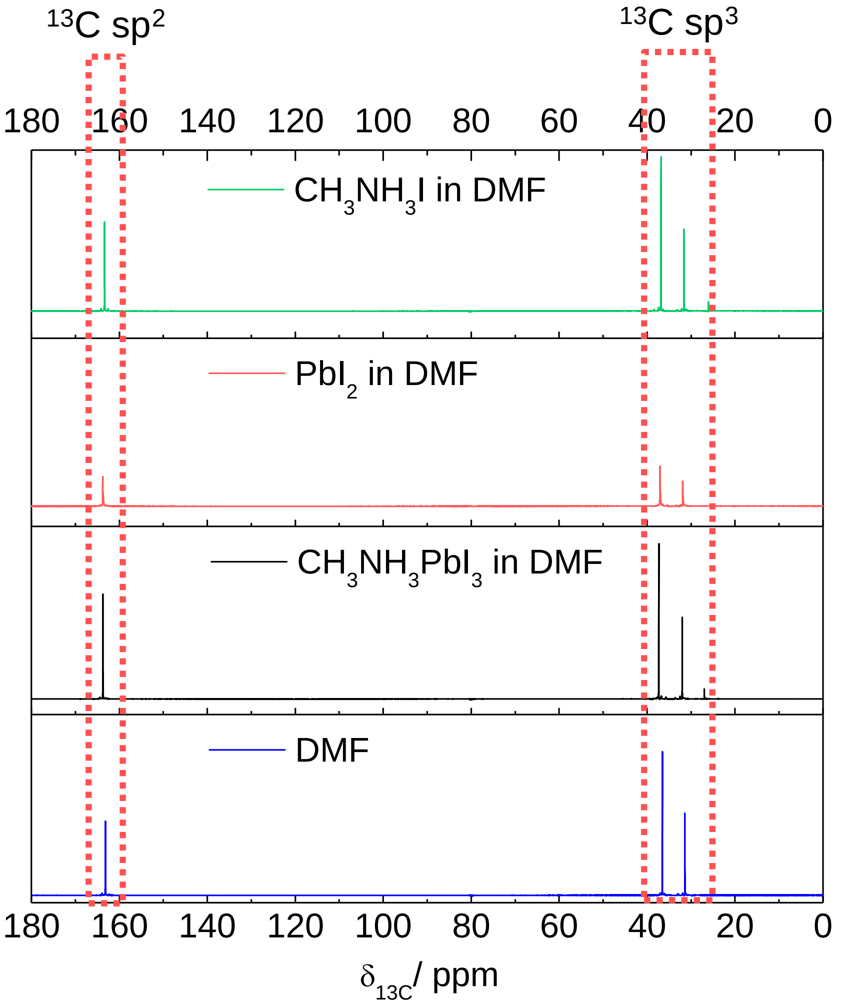

Supplementary Figure 5. 500 Mhz ^{13}C NMR spectra results. Full range of the ^{13}C NMR spectra of CH_3NH_3I-DMF, PbI_2-DMF, MALI-DMF and pure DMF solution. An expanded spectrum of the ^{13}C sp^2 and ^{13}C sp^3 range (Red-dot-square range) can be seen in Figs. 3a and 3b.

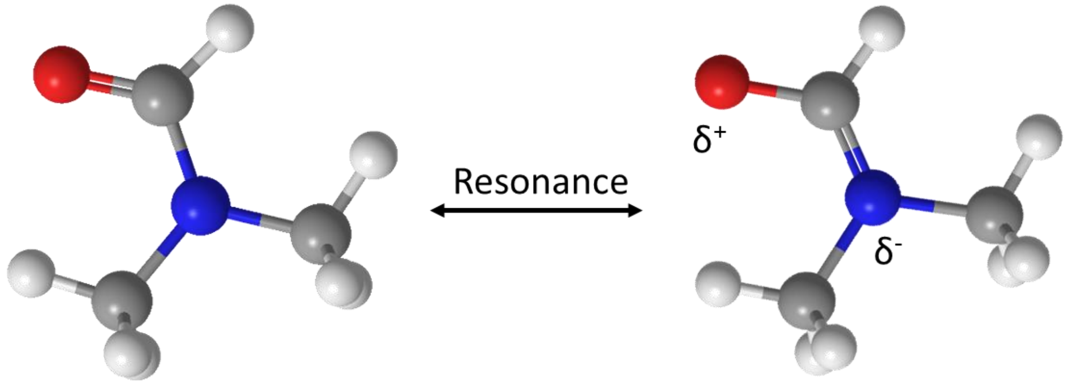

Supplementary Figure 6. Chemical resonance structure of DMF. A partially negative oxygen atom, which can bind to the Pb^{2+} of the perovskite material, is generated by the resonance structure of DMF (a polar aprotic solvent); red sphere: oxygen, blue sphere: nitrogen, gray sphere: carbon, white sphere: hydrogen

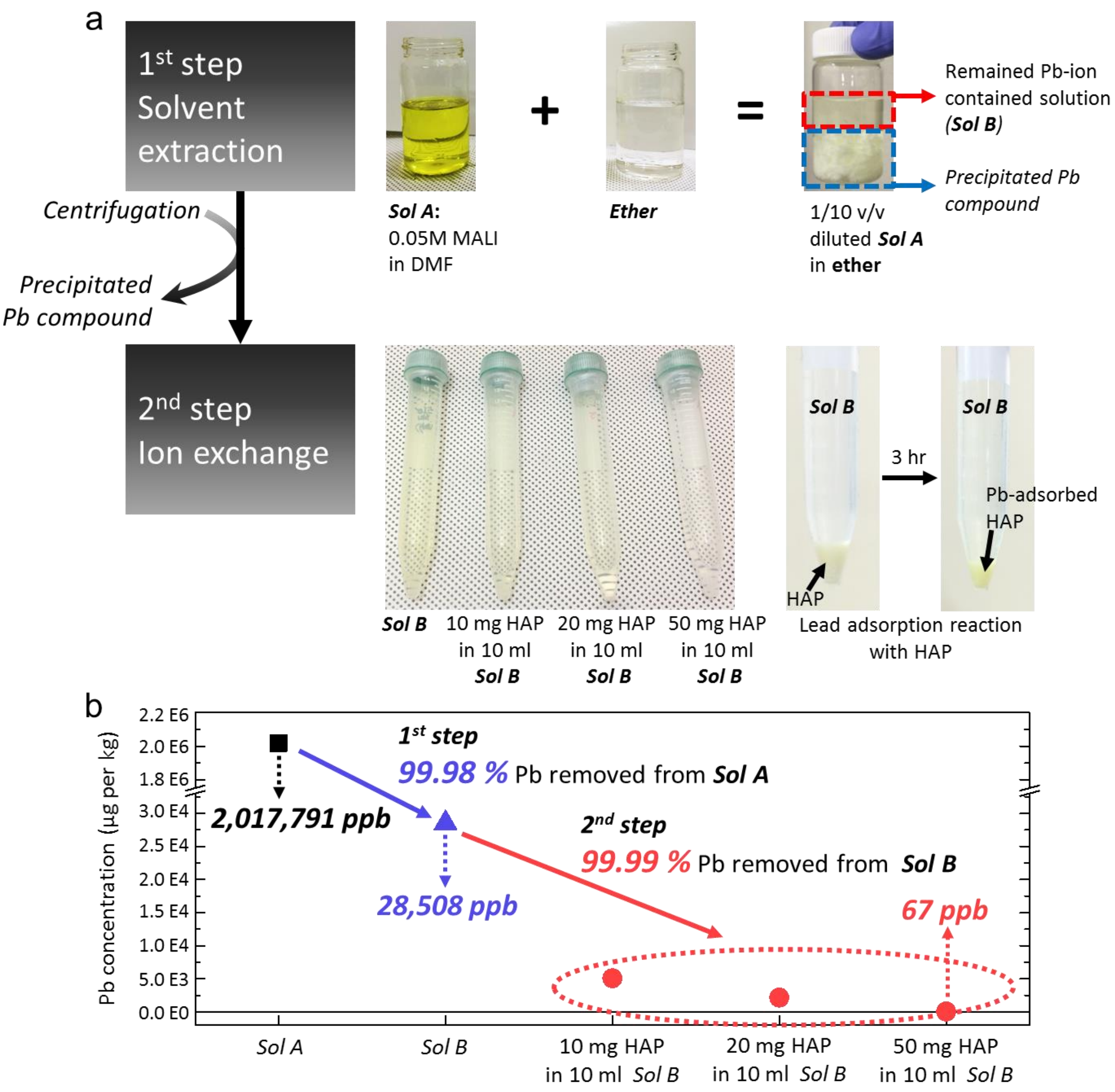

Supplementary Figure 7. 2 Steps of the lead removal process from residual-lead-containing recycling solvent. (**a**) Flowchart of the 2-step lead removal process and photos of each process. For this process, 0.05 M MALI in a DMF solution was prepared (Sol A). After the solvent extraction was performed, the precipitated lead compound was centrifuged, and the remaining solution (Sol B) was used for the ion exchange process. After the ion exchange process was complete, the added HAP caused a color change from white to light yellow. (**b**) The lead concentration of the solution obtained after each lead removal process.

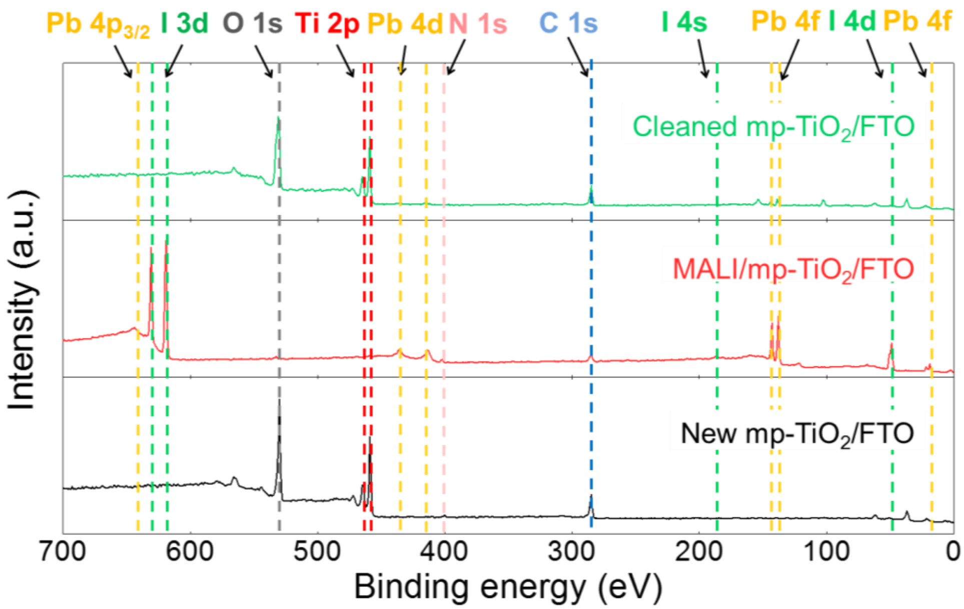

Supplementary Figure 8. Overview of the XPS spectra. Each XPS spectrum was calibrated using the C 1s core level. Using the same scale, new mp-TiO_2/FTO and cleaned mp-TiO_2/FTO XPS peaks show no significant difference, indicating that the Pb 4f and I 3d peaks in Figs. 4f and 4g are negligible.

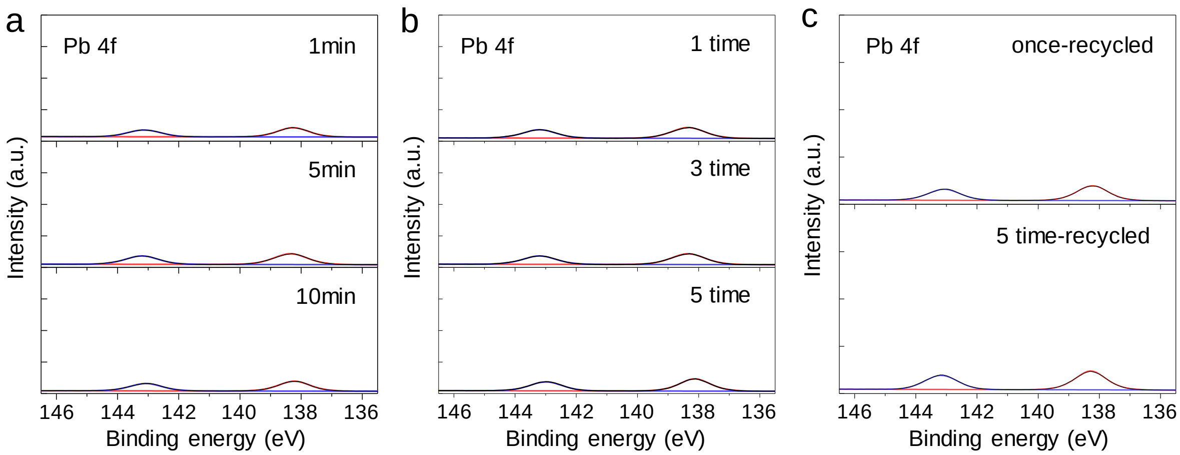

Supplementary Figure 9. XPS spectra of Pb 4f on cleaned mp-TiO_2/FTO substrates under various recycling conditions. XPS spectra of Pb 4f on cleaned mp-TiO_2/FTO with (**a**) various cleaning times (1, 5 and 10 min), (**b**) various numbers of cleaning cycles (1, 3 and 5 times with 30 s of cleaning in each cycle) and (**c**) once- and 5-times-recycled. To confirm the cleaning time, the number of cycles, and the accumulation effect of PbI_2, the XPS spectra of Pb are shown using the same y-axis scale as that in Figure 5g. The peaks located at 142.8 eV and 137.9 eV correspond well with the Pb elements in PbI_2. Each XPS spectrum was calibrated using the C 1s core level.

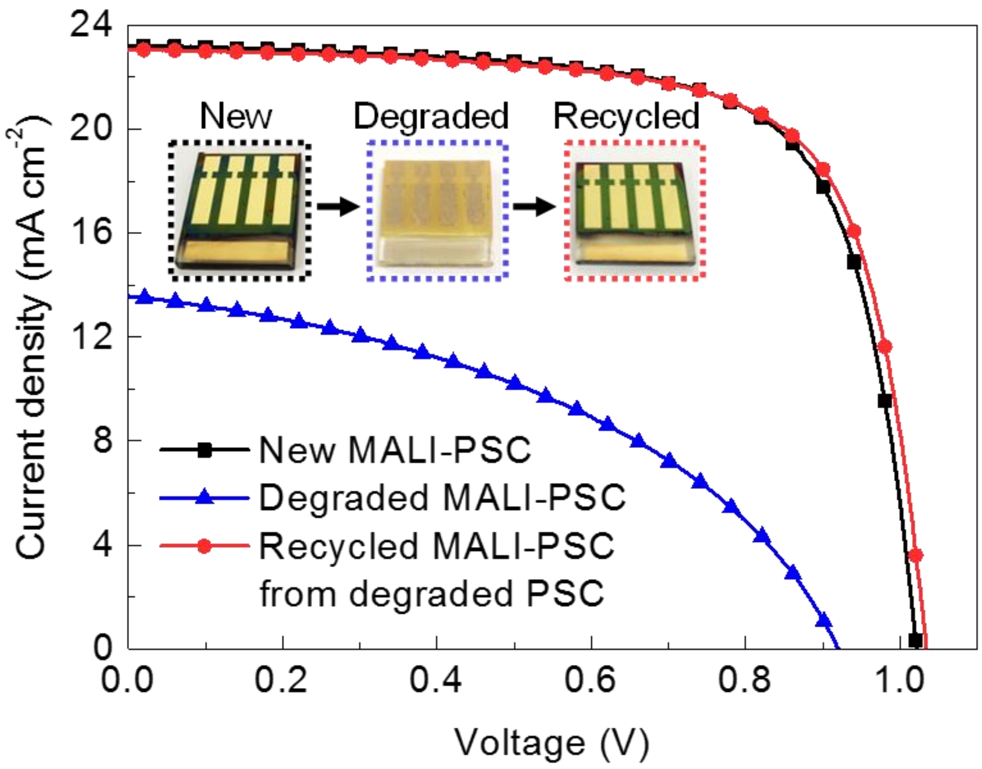

Supplementary Figure 10. *J-V* curve of new, degraded, and recycled MALI-PSCs. The photovoltaic performance of new, degraded, and recycled MALI-based PSCs. Inset photos show the color change of the devices, shown in the order that they were named. New PSC that was exposed to humidity levels above 99 % for 4 h was degraded. Recycled PSC was fabricated using a cleaned mp-TiO_2/FTO substrate that recycled a degraded PSC via the selective dissolution process.

`

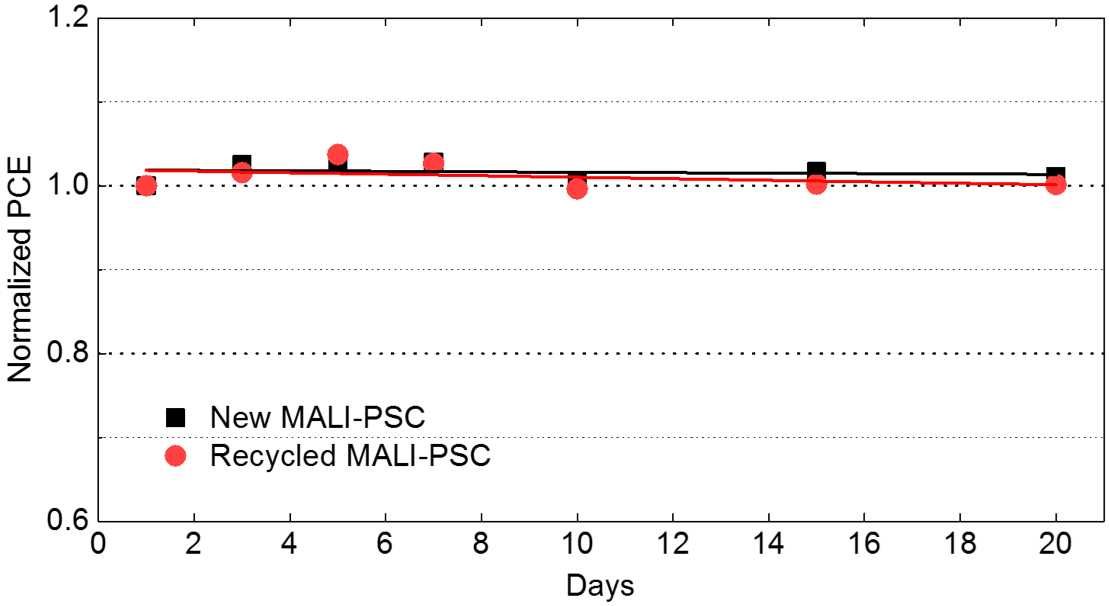

Supplementary Figure 11. Long-term stability comparison of MALI-based PSCs fabricated on the new and recycled mp-TiO_2/FTO substrate. The normalized power conversion efficiency (PCE) of MALI-PSCs fabricated on the new and recycled mp-TiO_2/FTO substrate. All devices were stored in the dark under ambient conditions.

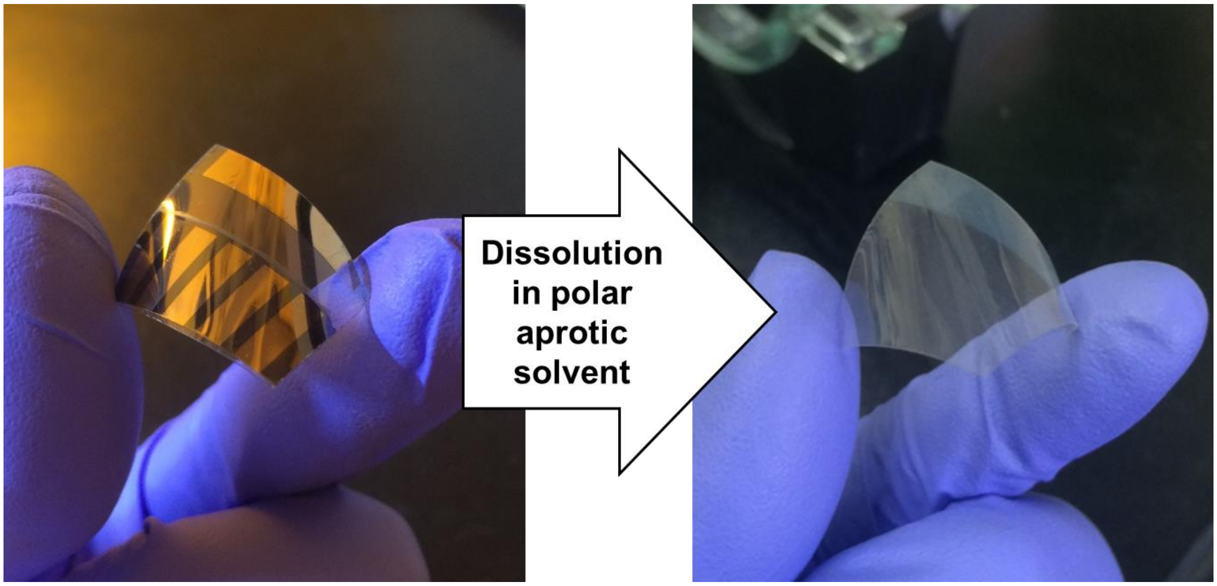

Supplementary Figure 12. Images before/after selectively dissolving flexible PSCs. The flexible TCG substrate can be recycled using the selective dissolving procedure. In this study, an Indium Tin Oxide/polyethylene naphthalate (ITO/PEN) flexible substrate was tested, and DMF was used as the solvent.

Supplementary Table 1. Concentration of elements measured using ICP-MS and the % of impurities of the collected gold electrodes. The concentration unit of the elements is ppb (equivalent to μg per kg).

Au	Pb	% of impurity in Au
2.981×10^6	1.214×10^4	0.41 %

Supplementary Table 2. Photovoltaic parameters of new and recycled PSCs over 20 days. The averages and standard deviations of the devices were calculated using 4 devices.

day	New substrate PSC				Recycled substrate PSC			
	J_{sc} (mA cm^{-2})	V_{oc} (V)	*FF*	PCE (%)	J_{sc} (mA cm^{-2})	V_{oc} (V)	*FF*	PCE (%)
1	22.81 ± 0.20	1.01 ± 0.02	0.70 ± 0.01	15.98 ± 0.50	22.89 ± 0.23	1.03 ± 0.02	0.68 ± 0.01	15.91 ± 0.76
3	22.71 ± 0.20	1.01 ± 0.02	0.71 ± 0.01	16.22 ± 0.45	22.79 ± 0.14	1.05 ± 0.01	0.69 ± 0.01	16.31 ± 0.2
5	22.72 ± 0.37	1.03 ± 0.02	0.71 ± 0.01	16.57 ± 0.55	22.79 ± 0.35	1.04 ± 0.02	0.69 ± 0.01	16.39 ± 0.76
7	22.50 ± 0.39	1.03 ± 0.02	0.71 ± 0.01	16.40 ± 0.64	22.38 ± 0.50	1.05 ± 0.01	0.69 ± 0.01	16.35 ± 0.54
10	22.33 ± 0.69	1.01 ± 0.02	0.70 ± 0.01	15.91 ± 0.90	22.43 ± 0.99	1.04 ± 0.02	0.69 ± 0.04	15.97 ± 1.68
15	22.49 ± 0.36	1.01 ± 0.01	0.70 ± 0.01	16.01 ± 0.32	22.61 ± 0.31	1.03 ± 0.03	0.69 ± 0.02	16.17 ± 0.91
20	22.65 ± 0.33	1.02 ± 0.02	0.70 ± 0.01	16.15 ± 0.63	22.57 ± 0.25	1.03 ± 0.01	0.68 ± 0.01	15.93 ± 0.25
